# Sickle cell trait in São Tomé e Príncipe: a population-based prevalence study in women of reproductive age

**DOI:** 10.1186/s12889-024-17761-1

**Published:** 2024-03-19

**Authors:** Guilherme Queiroz, Celdidy Monteiro, Licínio Manco, Luís Relvas, Maria de Jesus Trovoada, Andreia Leite, Celeste Bento

**Affiliations:** 1 Centro de Investigação em Antropologia e Saúde (CIAS), Universidade de Coimbra, Coimbra, Portugal; 2 NOVA National School of Public Health, Public Health Research Centre, Comprehensive Health Research Center, CHRC, NOVA University Lisbon, Av. Padre Cruz, Lisboa, 1600 − 560 Portugal; 3 Unidade de Saúde Pública, Unidade Local de Saúde da Região de Aveiro, Aveiro, Portugal; 4 Hospital Dr. Ayres de Menezes, São Tomé, São Tomé and Príncipe; 5 Departamento de Ciências da Vida, Universidade de Coimbra, Coimbra, Portugal; 6 Unidade Funcional Hematologia Molecular, Unidade Local de Saúde de Coimbra, Coimbra, Portugal; 7 Centro Nacional de Endemias, São Tomé, São Tomé and Príncipe; 8 Department of Epidemiology, Instituto Nacional de Saúde Doutor Ricardo Jorge (INSA), Lisbon, Portugal

**Keywords:** Sickle cell disease, Haemoglobinopathies, Genetics, Cluster sampling, Sub-saharian Africa

## Abstract

**Background:**

Sickle Cell Disorder is Africa’s most prevalent genetic disease. Yet, it remains a neglected condition, with high mortality under-five, and a lack of population-based studies in the region. This is the first of its kind in São Tomé e Príncipe, aiming to estimate the prevalence of sickle cell trait and other haemoglobin variants in women of reproductive age and its associated factors.

**Methods:**

We conducted a cluster survey in 35 neighbourhoods. Haemoglobin was assessed through point-of-care capillary electrophoresis or high-performance liquid chromatography, and sociodemographic data through questionnaires. The weighted prevalence of sickle cell trait (HbAS) and HbC carriers was estimated with a 95% confidence interval (95% CI). We calculated weighted prevalence ratios (95% CI) through robust Poisson regression for its association with age and individual and collective genetic heritage.

**Findings:**

The prevalence of sickle cell trait in women of reproductive age in São Tomé e Príncipe (*n* = 376) was 13.45% (95% CI: 9.05-19.00). The prevalence of HbC carriers was 8.00% (95% CI: 4.71-12.00). Older age and speaking Forro or Angolar were positively associated with having sickle cell trait.

**Interpretation:**

The prevalence of sickle cell trait in São Tomé e Príncipe ranks high in the West African region. The country should follow international guidelines, implementing newborn screening and comprehensive healthcare management.

**Supplementary Information:**

The online version contains supplementary material available at 10.1186/s12889-024-17761-1.

## Background

Sickle cell disorder (SCD) is an autosomal recessive condition, characterised by a mutation in the haemoglobin β-chain gene (*HBB:c.20 A > T*) that alters chain structures into Haemoglobin S (HbS). When subjected to hypoxia, HbS polymerises and forms fibrous precipitates, transforming the usual discoid, flexible red blood cell structure into a rigid, sickle-shaped structure [1]. Heterozygotic, sickle cell trait (SCT) is a benign condition assumed to confer protection against malaria and, therefore, an evolutionary advantage in endemic regions [2]. Contrarily, in homozygosity (HbSS) or compound heterozygotic forms (e.g. HbSC, HbSD, HbSbetatal), SCD is associated with high morbidity, vaso-occlusive crises with episodes of severe pain, anaemia, susceptibility to infection due to functional asplenia and ischaemic events [3].

Without proper clinical support, as observed in some of the African countries where it is most prevalent, the median survival of these patients is less than five years [4]. On the other hand, a prompt diagnosis and follow-up can reduce under-five mortality up to 10 times [5] with patients registering average life expectancies above 50 years [6]. Early case identification and implementation of comprehensive health care management is thus a fundamental strategy to tackle the disease and improve patients’ lives [7, 8]. The identification of parental SCT also allows timely genetic counselling when available. In addition to proven health gains, these strategies are highly cost-effective [9].

SCD is the most prevalent genetic disorder in Africa, where more than 1000 babies are born every day with the disease and 38,403 deaths from SCD were recorded in 2019, a 26% increase from 2000 [10]. In some sub-Saharan regions, SCT can be present in up to 30% of the population [2] and be responsible for more than 4% of all under-five mortality [11]. However, SCD has been constantly neglected by national and international agents, with insufficient funding and research on the disease and its impact and a lack of population-based studies [12]. São Tomé e Príncipe, the archipelagic country in the Gulf of Guinea, still lacks official numbers on carriers and patients. Three previous hospital-based studies point to an SCT prevalence between 13.00% [13], 14.10% (plus 1.92% of Hemoglobin C) [14] and 19.40% [15]. A revision of the 2021 Global Burden of Disease estimated that SCD was the 5th cause of under-five mortality in the country [16].

São Tomé e Príncipe has no newborn screening or other public health policy towards SCD. This is the first nationwide study in the country, and one of the first in Sub-Saharan Africa, aiming to estimate the prevalence of SCT and other haemoglobin variants among women of reproductive age and its associations with age and individual and collective genetic heritage. As a secondary objective, we intend to assess literacy on SCD (sickle cell knowledge) and its association with sickle cell status or relatives with SCD. We believe this can be an essential first step to designing future health plans to improve SCD patients in São Tomé e Príncipe and widen knowledge of the disease in Sub-Saharan Africa.

## Methods

### Study design and setting

This cross-sectional study was conducted in the country’s two islands, São Tomé and Região Autónoma do Príncipe, and consisted of an on-site collection of capillary blood and structured questionnaires. Data were collected between 10 and 30 April 2023; the remaining laboratory and statistical analyses were conducted in May of the same year.

### Participants

The study’s target population was women of reproductive age (15–49 years) living in São Tomé e Príncipe. We limited our analysis to this group as they would benefit the most from the results since the identified carriers could receive genetic counselling and test future offspring. As inclusion criteria, we considered women of this age, living in São Tomé e Príncipe. We excluded participants who had received a blood transfusion in the last three months, who were on a short or medium-term stay in the country (e.g. tourists, emigrants or expatriates), women who were sisters or mothers/daughters identified as such, or who had a previous clinical diagnosis of SCD and were referred to us by healthcare professionals to get the laboratory confirmation. In this last case, the test was conducted but we excluded them from the study to avoid a positive selection bias.

Due to the inability to conduct a nationwide simple random sampling, we employed a two-stage cluster sampling, with neighbourhoods as primary sampling units. The sampling process is detailed in Supplementary File 1.

### Study size

To calculate the sample size, we used the total number of women aged between 15 and 65 in São Tomé e Príncipe, identified in the 2012 Census: 48,983 women [17]. Assuming an expected frequency of 10.00%, consistent with previous studies, and for a 95% confidence interval (95% CI), this implied a minimum sample size of 138 women [18]. Based on analogous studies in the same region, we also assumed a design effect of 2.00 [19] and considered a refusal to participate rate of 10.00% [20]. Thus, we defined a minimum recruitment target of 304 women to achieve a minimum sample size of 276 participants. This value was employed in the clusters mentioned above.

### Data sources/measurements

We used a drop of blood to conduct capillary electrophoresis, using the point-of-care device Lab001 (ARKRAY Inc, Japan). Whenever it was not possible to perform this technique, we collected the samples on Guthrie filter paper, later analysed in Laboratório de Eritropatologia, at Centro Hospitalar e Universitário de Coimbra, Portugal, through high-performance liquid chromatography (HPLC) in the VARIANT II Haemoglobin Testing System (Bio-Rad Laboratories Inc., Hercules, CA, USA and through Multiplex Ligation-dependent Probe Amplification (MLPA) and Sanger sequencing to confirm dubious diagnoses.

Participants were also asked to answer a questionnaire. One team member read every question to ensure answers would not be affected by poor reading abilities, and always in the company of local volunteers who explained the questions if needed, avoiding interpretation issues and information bias.

### Variables

The main outcome was the woman’s *HBB* genotype, categorized as (1) “SCT” if she had a HbAS genotype, defined as a proportion of HbS between 20% and 45%; (2) “SCD” if she had an HBSS genotype, a pathological heterozygous combination of HbS with another haemoglobin variant (e.g. HbC, HbD), or an HBAS genotype if the proportion of HbA was below 30% (e.g. beta-thalassemia plus patients); (3) “HbAC” if she had a heterozygous combination of HbC and HbA, defined as a proportion of HbC between 20% and 45%; (4) “normal” if she had a HbAA genotype, defined as a proportion of HbA above 70% without any haemoglobin variant.

As independent variables, we collected sociodemographic characteristics of the participants: age, civil status, nationality, education level (knowing how to read or write) and past childbirths. As a proxy of collective genetic heritage, we asked if they spoke any local dialects: Forro, Angolar, Lunguie or Cape Verdian Creole. For individual genetic heritage, if they identified any relative with the disorder.

Quantitative variables were categorized into groups. Age was considered as seven intervals of five years (15–19 to 45–49); and pregnancies were considered as nulliparous (0), primiparous (1), multiparous (2–4) or grand multipara (> 4). Local dialects were also grouped as none, only Forro, only Angular and other combinations, including Lunguie. The variable of identifying any relative with SCD was grouped as (1) none; (2) a direct descendent, i.e. son(s) or daughter(s); (3) “ascendant 1” - father, mother, or sibling or nephew (as both sibling and nephew with SCD imply a direct ascendant with at least SCT); (4) “ascendant 2” - a grandparent or a first-degree uncle/aunt or cousin; and (5) second-degree uncle/aunt or cousin.

Finally, to assess of sickle cell knowledge, we used the naming of SCD (“bone disease” or sickle cell disease), the knowledge about the associated symptoms and how SCD was transmitted from one person to another.

### Statistical methods

All the statistical analyses were conducted using R version 4.1.0 [21]. 

First, we evaluated the suitability of the sampling design by calculating the intraclass correlation coefficient (ICC) using the R package “fishmethods” [22] and then the design effect, using the equation Deff = 1 + ICC(n– 1), where n is the average number of subjects sampled per cluster.

For sample characterisation, we calculated the absolute and relative frequencies. Even if the sampling was proportional to the size of each neighbourhood, and to address difficulties in recruitment, we adjusted the analysis considering each sample weight, calculated as the ratio between each neighbourhood population and the number of participants selected in that neighbourhood. The adjustment was conducted using the R package “survey” [23]. We then calculated the weighted prevalence of all the outcomes and the corresponding 95% CI. Haemoglobin proportions were represented using a boxplot to illustrate the dispersion of HbF in participants without Hb variants and with SCT, and of HbS in those with SCT.

The HW_TEST software [24] gave us the expected prevalence of each genotype based on the weighted tri-allelic frequencies, assuming a Hardy-Weinberg equilibrium (HWE) of the three alelles (A, S and C) at birth. We then tested the HWE with Chi-square without correction, and alpha set at 5%.

To assess if the age group, the spoken local dialect or an identified relative with SCD was associated with a higher risk of having SCT, we calculated the weighted prevalence ratio (PR) and corresponding 95% CI, through the robust Poisson method [25], using the “sandwich” package [26]. The association between a better knowledge of sickle cell and having SCT, or a relative with SCD, was also assessed using the same method.

## Results

A total of 376 women were enrolled in this study, representing 35 neighbourhoods of the seven health districts of São Tomé e Príncipe. Supplementary File 1 presents detailed information on neighbourhood selection and the number of participants per cluster. The ICC for the presence of HbS was 0.02, thus implying a Design Effect of 1.25. The anonymised dataset is available according to the requirements in the data statement.

**Table 1:** Characteristics of the participants: absolute and relative for both sample and (weighted) population

Variable	Group	N	Proportion (Sample)	Proportion (Weighted, 95% CI)
Age	15–19	29	7.71%	5.48% (2.48-10.00)
	20–24	54	14.36%	16.40%(12.80–21.00)
	25–29	60	15.96%	14.90% (9.60–22.00)
	30–34	77	20.48%	19.30% (14.10–25.00)
	35–40	65	17.29%	17.70% (13.60–22.00)
	40–44	42	11.17%	12.39% (8.58-17.00)
	40–49	49	13.03%	13.80% (10.30–18.00)
Civil Status	Single	311	82.71%	85.30% (79.20–90.00)
	Married	63	16.75%	14.53% (9.58-21.00)
	Other	2	0.53%	0.20% (0.03-1.00)
Nationality	São-Tomense	361	96.01%	96.40% (92.40–99.00)
	Cape Verdean (double)	13	3.46%	3.00% (1.12-7.00)
	Gabonese (double)	2	0.53%	0.44% (0.04-2.00)
Education	Knows how to read and write	352	93.62%	94.10% (90.00–97.00)
	Only knows how to read	13	3.46%	2.99% (1.11-6.00)
	Doesn’t know how to read or write	11	2.92%	2.88% (1.19-6.00)
Local dialect	None	101	26.86%	24.60% (19.20–31.00)
	Only Forro	158	42.02%	47.90% (36.80–59.00)
	Only Angolar	23	6.11%	4.08% (1.90-7.00)
	Only Cape Verdean	41	10.90%	8.26% (4.00–15.00)
	Other	53	14.10%	15.13% (9.96-22.00)
Past childbirths	Nulliparous (0)	55	14.63%	14.00% (11.0–17.00)
	Primiparous (1)	58	15.42%	18.10% (12.00–26.00)
	Multiparous (2–4)	205	54.52%	53.20% (45.90–60.00)
	Grand multipara (> 4)	58	15.42%	14.70% (10.90–19.00)

Table 1 presents sample and weighted frequencies. Most participants (67.98%) were between 20 and 39 years old. The low numbers registered below 19 years old were mainly due to the need for informed consent by the legal representative of underaged volunteers, usually absent at the collection site. Most women were single (82.71%), even though some advised they were partnered, as São Tomé e Príncipe polygamy and informal marriages are common practices. Most of them also knew how to read and write, and all of them were of São Tomense nationality, with a few having double nationality. The most spoken dialect was forro and around a quarter of the women only spoke Portuguese.

All the blood samples were processed successfully: 155 by point-of-care capillary electrophoresis and 221 by HPLC. The analysis of haemoglobin proportions according to sickle cell status for the point-of-care samples is presented in Supplementary File 2.

### Prevalence of SCT and other haemoglobinopathies

The weighted allele frequencies were 8.71% for the HbS allele and 3.99% for the HbC allele. Table 2 presents the prevalence of each genotype. The district with a higher proportion of participants with SCT was Região Autónoma do Príncipe, with 25.00% (5/20), followed by Lembá and Me-Zochi with 16.67% (5/30; 20/120), Água Grande with 14.17% (17/120), Caué with 13.33% (4/30), Cantagalo with 11.54% (3/26) and Lobata with 10.00% (3/30). The expected (weighted) prevalence of newborns with the HbSS genotype is 0.80% (0.40–1.20) and with the HbSC genotype is 0.70% (0.40-1.00). This means that between 8 and 22 of each 1000 newborns will develop SCD. The HWE could not be rejected (*p* = 0.30).

**Table 2:** Genotype: absolute and prevalence in the sample and in the population (weighted)

Genotype	N	Prevalence (Sample)	Prevalence (Weighted, 95% CI)
HbAA	301	80.05%	77.20% (68.30–85.00)
HbAS	49	13.03%	13.45% (9.05-19.00)
HbAC	18	4.79%	6.79% (3.77-11.00)
HbSS	2	0.53%	1.38% (0.25-4.00)
HbSC	6	1.60%	1.20% (0.31-3.00)

Additionally, we tested two participants with an HbAA genotype that presented levels of Foetal Haemoglobin (HbF) above 12%. One had a heterozygous mutation known as Cape Verdean deltabeta deletion [27]and the other a frameshift (HBB:c.126-129delCTTT).

### The individual and familiar history of SCD

More than half of the participants (63.03%) didn’t know whether any relative had SCD. Among the 139 recognizing at least one relative with the condition, 32.37% had a grandparent or first-degree uncle or aunt, 39.57% had the mother, father, sibling, or nephew, 13.67% had a son or daughter and 10.07% had a second-degree uncle or aunt. There were also 15 participants reporting that they suffered from SCD. Of these, one had HbS in homozygosity, three had SC haemoglobinopathy, six only had SCT, and five had no single haemoglobin variant of interest. Though we do not ask why the latter assumed the diagnoses, we presume it was due to either a false positive sickling test or to clinical misdiagnoses of pain syndromes.

### Factors associated with SCT

**Fig. 1:**
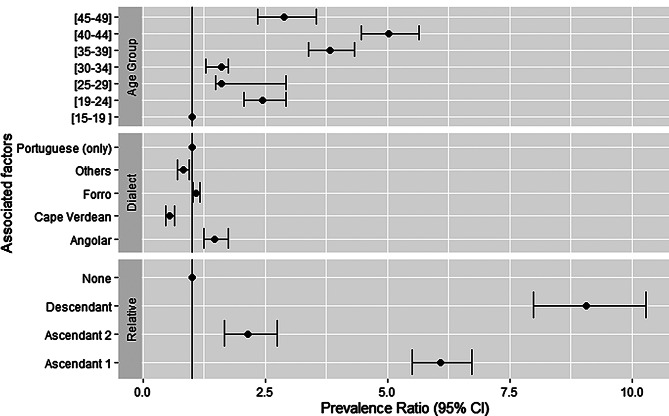
Prevalence ratios for associations of age, dialect and relative with SCD with having SCT. PR– Prevalence Ratio. CI– Confidence Interval. Descendant - son or daughter; Ascendant 1 - father, mother, sibling or nephew; Ascendant 2 - grandparent or first-degree uncle, aunt or cousin. Reference classes are presented as PR of 1.

Figure 1 presents prevalence ratios of SCT according to age, dialect and having a relative with SCD. Age was significantly associated with having SCT, especially in the groups above 30 years old. The participant’s dialect registered different associations when compared to those who only spoke Portuguese. Speaking Forro or Angolar increased the prevalence by 9.29% (95% CI: 2.35–16.70) and 46.86% (95% CI: 23.54–73.88) respectively while speaking only Cape Verdean or other combinations lowered the prevalence by 55.12% (95% CI: 46.74–65.02) and 82.30% (95% CI: 71.35–94.93). Compared to individuals without any known relatives with SCD, those who had a son or daughter showed the highest probability of having SCT, with a prevalence 806.22% (95% CI: 699.20-925.60) ahigher than the reference group. This probability decreased by a third when the individual only had a mother, father, sibling, or nephew, and remained 113.95% (95% CI: 67.07-173.98) higher in other first-degree relatives.

### Sickle cell knowledge

When asked about their knowledge of SCD, only seven participants did not recognize the condition. The most used name was sickle cell disease, “cell disease” or just “cell” (69.65%), 45.26% named it “bone disease” and 14.90% would use both names. As for signs and symptoms, most women associated the disease with pain (79.25%), 14.36% with fatigue and 6.65% with anaemia. Knowledge of the transmission of SCD was scarcer, with 63.56% admitting they didn’t know it and just 28.99% answering it was hereditary, from both mother and father.

When analysing the influence of each sickle cell status on sickle cell knowledge, we observe that people with SCT were 61.48% (95% CI: 59.54–63.48) less probable of being ignorant of SCD transmission, but 131.82% (95% CI: 98.70-170.47) more potential of being unaware of its symptoms. On the other hand, those without relatives with SCD were 67.16% (95% CI: 59.34–75.36) and 148.69% (95% CI: 99.52-209.98) more probable of being ignorant of both transmission and symptoms, respectively.

## Discussion

Our study estimated an SCT prevalence of 13.45% (95% CI: 9.05-19.00) among women of reproductive age in São Tomé e Príncipe. Due to its autosomal condition, we don’t expect a different prevalence of SCT according to sex [28]. Thus, we expect that this value corresponds to the estimated prevalence of SCT in the whole population between 15 and 49 years in São Tomé e Príncipe. This prevalence is accordant to the previous studies in the country [13–15]. Ranking high in the interval of prevalence found in West Africa [2], this highlights the need for São Tomé e Príncipe to participate in the regional effort to tackle the disease [12].

The prevalence of the HbC carriers, 6.79% (95% CI: 3.77-11.00), though higher than the previously recorded, is the first recorded in a representative sample. The value aligns with the ones observed in neighbouring countries [29]. When looking at the HbSS genotype alone, we also obtained a prevalence − 1.38 (95% CI: 0.25-4.00) - comparable to that of the region [30]. In any case, these are only indicative values, as the study design only considered SCT prevalence. However, they show that future newborn screening strategies should recognize HbC and beta-thalassemia.

The positive association of SCT with age has been previously reported [28] and may be related to its protection against malaria, still endemic in the country, increasing long-term survival. As malaria elimination in São Tomé e Príncipe gets closer, these differences may disappear with time. However, the possibility of an HWE does not reflect the expected survival bias favouring the HbA allele frequency. This may imply a slight positive bias in the selection of SCD patients or an eventual balance between the negative survival bias of SCD and the positive one in SCT (malaria-related). Further research on comparative mortality between genotypes would be useful to clarify this.

Forro and mainly Angolar-speaking people had higher SCT prevalence, while the ones that spoke Cape Verdean creole or combinations of dialects had lower prevalence. This supports the theory of different genetic lineages according to the geographical origin of each ethnic group, as these findings are in line with the lower prevalence found in Cape Verde and higher in the Gulf of Guinea and Angola. On the other hand, it also suggests a genetic difference between Tonga (Cape-Verdean descendants) and Forros which was previously discarded [31]. Studies on the genetic diversity of São Tomé e Príncipe would be required to comprehend the history of its colonisation better.

The overall recognition of SCD was impressive, with most correctly naming the disease and identifying pain as a symptom. It would be useful for future campaigns to clarify the “bone disease” nomenclature and avoid eventual misdiagnoses of osteoarticular syndromes or dengue. Most of the participants correctly identified the transmission of the disease as hereditary, showing that there is a literacy base for health strategies addressing genetic counselling. The positive association of sickle cell status with sickle cell knowledge reveals a vulnerability of the target population to those strategies. The opposite was noted for people without close relatives with SCD and may imply that people without close contact with the disease need to be better informed about SCD. Since previous studies did not include the recognition of signs and symptoms [32], it would be helpful to include these variables in future sickle cell knowledge scales.

The lack of recent demographic data on São Tomé e Príncipe is a limitation of this paper, as current estimates point to a population that almost doubled the one in the last census [33]. This means that the distribution used in the sampling procedure may not correspond to the current one. However, the primary demographic flux was towards the capital (Água Grande district), already overrepresented in our sample as this tendency was already present in 2012.

Besides, when analysing age distribution in our sample, we realised that younger groups are underrepresented since they constitute most of the population but not of our sample. This may be related to the nonrandom selection in the second stage of sampling and the use of schools or health services as collection sites, not frequently used by women this age. Although that may have influenced each location’s results, the collection points choice tended to overcome this bias. This limitation may hinder our population-based estimates, yet we attempted to minimise it using weighted analyses.

Haemoglobin analysis was conducted with highly sensitive and specific tests to avoid misclassification. Data on literacy may also have been subjected to social-desirability bias, but we tended to avoid replications of answers among participants by controlling the interview settings.

## Conclusion

The data supports that SCD is a significant health problem in São Tomé e Príncipe that shall be addressed by national strategies such as neonatal screening, updating therapeutic guidelines and capacitation of healthcare workers. Neighbouring countries with lower prevalence of SCT have already addressed the issue, which was also set as a health priority by the Ministers of Health of the Region [10]. Future research on SCD in the country should also focus on younger generations, the quality of life and life expectancy of SCD patients, to better design support strategies. Data shows that there’s a solid base of knowledge encouraging future programmes that involve the community. São Tomé e Príncipe, with its high prevalence of SCT, its scale, organization and culture, has the opportunity to be an example in Sub-Saharan Africa and significantly improve the lives of patients.

### Electronic supplementary material

Below is the link to the electronic supplementary material.

**Supplementary Material 1:** Cluster sampling

**Supplementary Material 2:** Hemoglobin proportion analysis

## Data Availability

De-identified data and study protocol can be requested by e-mail to the corresponding author (GQ) and depends on the authorization of Centro de Investigação em Antropologia e Saúde and Centro Nacional de Endemias da República Democrática de São Tomé e Príncipe. The request must include a methodologically sound proposal with the full study protocol. All requests will be evaluated individually by both institutions, with data being available until 5 years following the publication. All shared data will ensure that the rights and privacy of the participants will be safeguarded during the process. The model code is available at https://gitlab.com/drepa-comunidade/sickle-cell-trait-in-stp.

## Abbreviations

CI: Confidence Interval
HWE: Hardy-Weinberg equilibrium
ICC: Intraclass Correlation Coefficient
HbA: Haemoglobin A
HbC: Haemoglobin C
HbF: Faetal Haemoglobin
HbS: Haemoglobin S
HPLC: High-Performance Liquid Chromatography
MLPA: Multiplex Ligation-dependent Probe Amplification
PR: Prevalente Ratio
SCD: Sickle Cell Disorder
SCT: Sickle Cell Trait
