# Supplementary material for: Sickle cell trait in São Tomé e Príncipe: a population-based prevalence study in women of reproductive age
Source: BMC Public Health. 2024 Mar 19;24:850. doi: 10.1186/s12889-024-17761-1 (PMC10949760; doi:10.1186/s12889-024-17761-1)
Supplement: Supplementary file 1 — Supplementary Material 1: Cluster sampling [file 12889_2024_17761_MOESM1_ESM.docx]

**Supplementary File 1 - Cluster Sampling**

**Methods:**

As primary sampling units (PSU), we used the census units’ “neighbourhoods”, as reported by the National Institute of Statistics (INE) of São Tomé and Príncipe. In the first stage, we randomly selected 35 neighbourhoods according to a probability proportional to their population size. For this purpose, we considered the female population between 15 and 65 years old of each neighbourhood published in the 2012 Census. Neighbourhoods with less than 50 people were excluded to ensure a minimum number of participants. Using Microsoft Excel, we listed the eligible neighbourhoods in alphabetical order. We calculated the cumulative population along this list, assigning each neighbourhood a number corresponding to the cumulative population achieved in its position. A number was then randomly drawn between 1 and the number assigned to the last neighbourhood, i.e. the total population considered, and the neighbourhood where it was achieved was selected and removed from the list until reaching a total of 35 selected neighbourhoods. This approach ensured that more populated neighbourhoods would have a higher probability of selection (probability proportion to size).

In the second stage, 10 participants were targeted in each neighbourhood, yielding a target number of 350 women. For technical reasons, such as electricity access, and to guarantee the legitimation of the study among the community, interviews and samples were collected at fixed points in the neighbourhoods, like health centres, schools, or community centres. In dense urban areas with an intense circulation of people near adjacent neighbourhoods, we selected a common reference point that assisted people from the selected neighbourhoods (*e.g.* school, health post…). All collection points are listed in Table 2. To avoid selection bias, the survey was not announced by any means; whenever required, it was previewed with the responsible person for the collection point. In most cases, improvised volunteers would ask women to participate in the study as they walked by, and it was impossible to register refusals as initially considered. However, our overall perception was that adherence was near 100%.

**Table 1: Characteristics of the selected neighbourhoods, collection point used and number of samples collected.**

| **Neighbourhood** | **District** | **Population (2012)** | **Collection Point** | **N** |
| --- | --- | --- | --- | --- |
| Agostinho Neto | Lobata | 992 | Restaurant (Jardela) | 10 |
| Bela Vista | Lobata | 517 | Bela Vista Community Center | 10 |
| Maianço Roça | Lobata | 209 | Roça Mainço Community Center | 10 |
| Benga | Lembá | 3589 | Lembá Health Center | 10 |
| Diogo Vaz | Lembá | 632 | Diogo Vaz School | 10 |
| Rosema | Lembá | 2587 | Lembá Health Center | 10 |
| Água Lama | Mezochi | 336 | Bom Bom Health Center | 10 |
| Aldeamento Monte Café | Mezochi | 194 | Monte Café Hospital | 10 |
| Batepá | Mezochi | 775 | Trindade Hospital | 10 |
| Cabalo Molê | Mezochi | 425 | Trindade Hospital | 10 |
| C. Trindade Centro | Mezochi | 1586 | Trindade Hospital | 10 |
| Melhorada | Mezochi | 656 | Trindade Hospital / Caixão Grande Health Center | 20 |
| Praia Melão | Mezochi | 2668 | Bom Bom Health Center | 10 |
| Riba Mato | Mezochi | 363 | Bom Bom Health Center | 10 |
| Santa Margarida | Mezochi | 384 | Santa Margarida School | 10 |
| Uba Flor | Mezochi | 588 | Trindade Hospital | 10 |
| Bem Posta | Mezochi | 120 | Monte Café Hospital | 10 |
| Água Arroz | Água Grande | 2238 | Água Arroz Health Center | 10 |
| Almeirim | Água Grande | 1591 | Água Arroz Health Center | 10 |
| Boa Morte | Água Grande | 3432 | Boa Morte School | 10 |
| Hospital | Água Grande | 1881 | Hospital Community Center | 10 |
| Liberdade | Água Grande | 512 | Liberdade School | 10 |
| Madre de Deus | Água Grande | 2469 | Madre de Deus Health Center | 10 |
| Oquê-del-Rei | Água Grande | 3465 | Liberdade School | 10 |
| Praia Cruz | Água Grande | 1652 | Praia Gamboa Health Center | 10 |
| São João da Vargen | Água Grande | 1793 | Vila Fernanda Health Center | 10 |
| São Marçal | Água Grande | 2866 | Bom Bom Health Center / São Marçal Health Center | 20 |
| Vila Fernanda | Água Grande | 802 | Vila Fernanda Health Center | 10 |
| Água Izé | Cantagalo | 1255 | Água Izé Health Center | 16 |
| Cidade Santana Centro | Cantagalo | 769 | Santana Health Post | 10 |
| Beira Mar | Caué | 510 | São João dos Angolares School | 10 |
| Meven Ngay | Caué | 406 | São João dos Angolares School | 10 |
| Porto Alegre | Caué | 795 | Centre for Educational and Training Resources (CREF) | 10 |
| Lenta-Piá | RA Príncipe | 1020 | Santo António Hospital | 11 |
| Sundy | RA Príncipe | 416 | Sundy Health Center | 9 |
