# Supplementary material for: Sickle cell trait in São Tomé e Príncipe: a population-based prevalence study in women of reproductive age
Source: BMC Public Health. 2024 Mar 19;24:850. doi: 10.1186/s12889-024-17761-1 (PMC10949760; doi:10.1186/s12889-024-17761-1)
Supplement: Supplementary file 2 — Supplementary Material 2: Hemoglobin proportion analysis [file 12889_2024_17761_MOESM2_ESM.docx]

**Supplementary File 2 - Haemoglobin proportion analysis**

Figure 1 depicts the distribution of the Hb variants in the sample studied with electrophoresis. This analysis excludes SCD cases (HbSS and HbSC) as the equipment was not able to give Hb proportion in the absence of HbA. Participants with SCT presented HbS proportions between 21.8% and 33.8%, with a mean of 29.0% (sd 3.0). HbC carriers presented similar values for HbC, with proportions between 27.0% and 34.8%, with a mean value of 32.0% (sd 2.84). In both SCT and HbC carriers, there was an overlapping of HbA proportion, with mean values of 58..4% (sd 2.6) and 58.0% (sd 2.3) respectively. On its side, HbF registered similar distributions between the three groups, with SCT cases registering slightly higher values - a mean value of 1.14% (sd 0.81) against 0.79% (sd 0.50) for HbC carriers and 0.99% (sd 1.34) for participants with no variant. Levels of HbF above 2.0% were observed in 15 participants, three with SCT and 12 without HbS or HbC, one of them (no SCT) with 12.8%.

**Figure 1: Hb variants proportion according to sickle cell status**


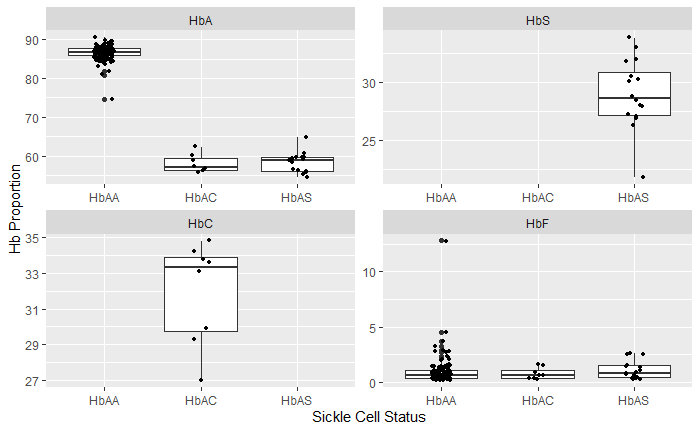


**Interpretation**

The proportion of HbS in SCT is affected by the association with alpha thalassemia. HbS levels can be lower in the case of concomitant heterozygous alpha thalassemia, and much lower in the homozygous state. As the -alpha 3.7 deletion is a benign condition frequent in African countries, the lower values of HbS registered may be caused by double heterozygosity with the -alpha 3.7 deletion.

The high levels of HbF (> 2.0%) registered in some of the samples are not relevant, except for the two samples with more than 12.0%. One had a heterozygous mutation known as Cape Verdean deltabeta deletion, and the other had a frameshift (HBB:c.126-129delCTTT).
